# A novel approach to predicting the synergy of anti-cancer drug combinations using document-based feature extraction

**DOI:** 10.1186/s12859-022-04698-8

**Published:** 2022-05-05

**Authors:** Yongsun Shim, Munhwan Lee, Pil-Jong Kim, Hong-Gee Kim

**Affiliations:** 1 Biomedical Knowledge Engineering, Seoul National University, Seoul, Republic of Korea; 2 School of Dentistry and Dental Research Institute, Seoul National University, Seoul, Republic of Korea

**Keywords:** Anti-cancer drug combination, Drug synergy, Document-based feature extraction, Text mining, Natural language processing, Word2vec, Deep learning, Machine learning

## Abstract

**Background:**

To reduce drug side effects and enhance their therapeutic effect compared with single drugs, drug combination research, combining two or more drugs, is highly important. Conducting in-vivo and in-vitro experiments on a vast number of drug combinations incurs astronomical time and cost. To reduce the number of combinations, researchers classify whether drug combinations are synergistic through in-silico methods. Since unstructured data, such as biomedical documents, include experimental types, methods, and results, it can be beneficial extracting features from documents to predict anti-cancer drug combination synergy. However, few studies predict anti-cancer drug combination synergy using document-extracted features.

**Results:**

We present a novel approach for anti-cancer drug combination synergy prediction using document-based feature extraction. Our approach is divided into two steps. First, we extracted documents containing validated anti-cancer drug combinations and cell lines. Drug and cell line synonyms in the extracted documents were converted into representative words, and the documents were preprocessed by tokenization, lemmatization, and stopword removal. Second, the drug and cell line features were extracted from the preprocessed documents, and training data were constructed by feature concatenation. A prediction model based on deep and machine learning was created using the training data. The use of our features yielded higher results compared to the majority of published studies.

**Conclusions:**

Using our prediction model, researchers can save time and cost on new anti-cancer drug combination discoveries. Additionally, since our feature extraction method does not require structuring of unstructured data, new data can be immediately applied without any data scalability issues.

## Background

Cancer is a global health threat with a high mortality rate; thus, intense research efforts are ongoing to develop new anti-cancer drugs [1]. The development of new anti-cancer drugs follows two approaches: drug discovery, developing previously undiscovered drugs [2], and drug repositioning, discovering drugs with anti-cancer effects among drugs used for other diseases [3]. In addition to these approaches, the importance of drug combination studies, combining two or more drugs, is increasing [4].

Two main aims lie behind drug combinations: minimizing drug side effects by reducing drug dosage and achieving a higher therapeutic effect than that of a single drug [5]. In drug combination research, synergy means that the therapeutic effect is increased by combining two or more drugs [6]. For example, the AZD/crizotinib combination has proven effective in both in-vivo and in-vitro cancer treatment experiments [7]. To discover the optimal drug combination, various conditions must be considered such as the side effects between drugs and the dosage of each drug. The number of drug combinations increases exponentially with regards to the total drug amount and dosages per drug.

Drug combinations are discovered in in-vivo and in-vitro experiments. Recently, high-throughput screening has been applied to drug combination discovery, but discovering and filtering potential cases by this method is time-consuming and expensive. To resolve this problem, researchers used in-silico methods, which analyze large amounts of data to discover hidden and meaningful patterns [8]. In-silico methods discover potential drug combinations by extracting them from biomedical data through machine learning.

Using a chemical and genomic information-based normalization strategy, Preuer et al. identified heterogeneity in the input data and created DeepSynergy, a deep learning based drug synergy model using conical layers [9]. Xia et al. presented a neural network-based computational model to encode molecular feature types (gene expression, microRNA, and proteome) and predicted cell line responses to a subset of drug pairs [10]. Kim et al. presented a drug synergy prediction model based on a multitask deep neural network using molecular and genomic drug features, as well as type and genomic cell line features [11]. Zhang et al presented AuDNNsynergy, a deep learning-based drug combination prediction model with encoded multi-omics data (gene expression, mutation, copy number, etc.) using an autoencoder [12]. Janizek et al. presented TreeCombo, an XGBoost-based drug combination prediction model using chemical and physical drug features and cell line gene expression levels [13]. Celebi et al. proposed a model predicting biologically relevant synergistic drug combinations and cell line features using XGBoost [14]. Jeon et al. presented a personalized drug combination prediction model based on extremely randomized trees(ERT) using genomic information, drug targets, and pharmacological information [15]. Li et al. presented a logistic regression-based drug combination prediction model using cell line features (gene expression and essentiality) and drug features (drug target information) [16].

In most published studies, drug and cell line features were extracted using structured data. Since structured data is mostly created via manual curation, they cannot be easily applied immediately, making data expansion difficult. Unstructured data, such as biomedical documents, include experimental types, methods, and results [17]. By using this information, the unique characteristics of drugs and cell lines can be expressed, and new relationships between drugs and cell lines can be discovered. Therefore, it can be potentially beneficial to predict anti-cancer drug combination synergy using extracted features from documents. However, few studies predict anti-cancer drug combination synergy by extracting features from documents. To solve this problem, we constructed drug and cell line features using biomedical documents. Since our approach uses unstructured data, few restrictions apply with regards to data scalability.

In this study, we propose a novel approach to predicting the synergy of anti-cancer drug combinations using features extracted from documents. The prediction model was created by applying various deep learning and machine learning methods used in published studies, and the results were compared. The model using our features were achieved higher result than majority of published studies. The results show that our approach has promise compared to other feature extraction methods of published studies. Based on the results, researchers can apply our prediction models to predict whether an anti-cancer drug combination has synergy in a specific cell line before in-vivo or in-vitro experiments.

## Methods

**Fig. 1:**
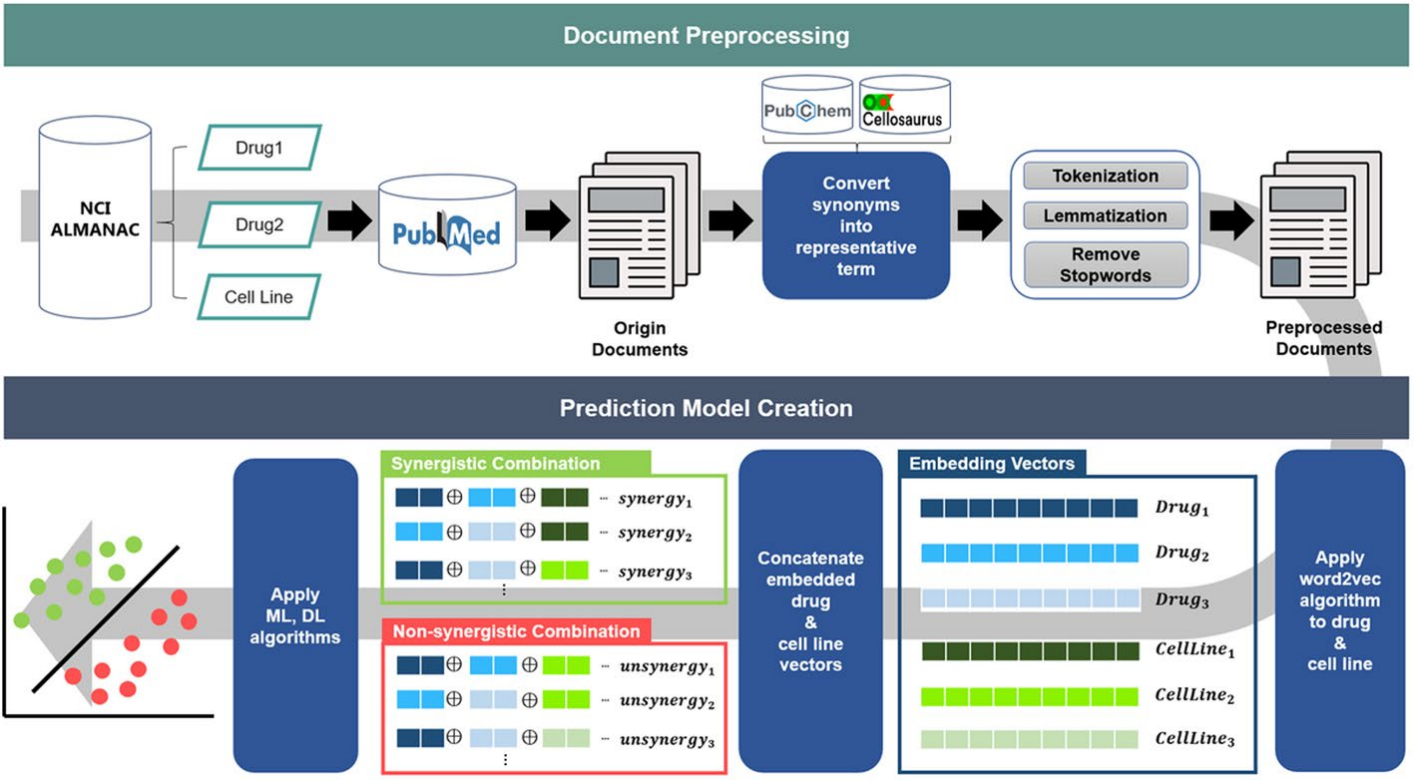
Overview of our approach

The process is divided into two steps. First, for document preprocessing, we extracted documents containing keywords of validated anti-cancer drug combinations and target cell lines. Then, the extracted documents were preprocessed. Second, in the prediction model creation process, drug and cell line features were extracted using preprocessed documents, and training data were constructed by concatenating the features. Then, a deep and machine learning-based prediction model was created by performing a 5-fold cross validation of the combinations. Figure 1 shows the overall workflow. The details of each step are as follows.

### Document preprocessing

A large matrix of anti-neoplastic agent combinations(ALMANAC) is the most widely used golden standard data in anti-cancer drug combination research, and it was presented by the National Cancer Institute (NCI) [18]. NCI-ALMANAC has been constructed as a drug dose-response matrix by administering more than 5000 FDA-approved drug combinations to 60 human tumor cell lines. Additionally, ComboScore, the reactivity result of each combination, was calculated based on the bliss [19] score.

The PubMed database is a repository of biomedical documents. We extracted publications from PubMed containing drug and cell line keywords of the NCI-ALMANAC. As PubMed contains a variety of biomedical publications with no specific format, the same drugs and cell lines are differently marked in the documents. For example, in PubMed id “10026447”, the drug Fluorouracil is written as Fluorouracil, whereas in “10026446” it is written as 5-FU. Fluorouracil and 5-FU represent the same drug, but the labels are different depending on the publication. Therefore, the information on the same drug is dispersed, hindering feature extraction. To resolve this problem, we converted synonyms in documents into representative words. When converting synonyms into representative words, PubChem [20] and Cellosaurus [21] were referred to for drug and cell line words, respectively. Next, we separated the words through tokenization in documents, performed lemmatization, and removed stopwords.

### Prediction model creation

The Word2vec algorithm is a method for embedding a target word using surrounding words [22]. Therefore, all information in the text can be used for embedding features of drugs and cell line. This is an advantage of using unstructured data such as documents when performing feature extraction. In this step, we extracted document-based features by embedding drugs and cell lines from the preprocessed documents. Table 1 shows the hyperparameter settings used to create the Word2vec model.

**Table 1:** Hyperparameter setting of Word2vec

Hyperparameter name	Value
Vector size	256
Window	5
Min count	1
sg	0
Epochs	200

Since ComboScore is calculated according to drug concentration in NCI-ALMANAC, we used the average combination ComboScores. Table 2 provides an example of NCI-ALMANAC with the average. We separated the dataset into synergistic and non-synergistic combinations using the average combination ComboScores. We extracted the embedding vectors of drug and cell line terms in NCI-ALMANAC from the Word2vec model, and constructed the training data by concatenating drug and cell line features based on synergistic and non-synergistic combinations. A prediction model based on deep learning and machine learning algorithms that can classify various classes was created using the training data. The deep learning-based model used feed-forward neural network (FFNN) [23] and autoencoder (AE) [23], and the machine learning-based model used XGBoost (XGB) [24], extremely randomized trees (ERT) [25], and logistic regression (LR) [26]. FFNN is a network model in which several layers of perceptrons are sequentially pasted and connected in the direction of the input layer, the hidden layer, and the output layer. AE is a deep neural network model that compresses and reduces input data, then expands it again to make the resulting data identical to the input data. XGBoost is an ensemble algorithm that combines several decision trees and delivers the best performance in tree boosting. ERT is a modified form of the random forest model, which increases randomness by randomly dividing candidate attributes of each forest tree. LR is a model that classifies data into discrete classes for a given feature. We created a prediction model that can classify the synergy of anticancer drug combinations using each learning model. Kunjie et al. constructed a unified framework that can use computational models or architectures presented in published studies [27]. Generally, direct comparison tests between studies are difficult as the data and algorithms used in each study are different. Using their framework, we directly compared each of the prediction models using the same dataset. In addition, since the framework used the same hyperparameters as the published studies, we created the prediction model using the default settings.

**Table 2:** Examples of NCI-ALMANAC

Drug1	Drug2	Cell line	ComboScore
Methotrexate	Hydroxyurea	SF-295	14.22
Busulfan	2-Fluoro Ara-A	CAKI-1	14.33
Azacitidine	Thiotepa	NCI-H460	20.44
Methotrexate	Dactinomycin	786-0	− 7.22
Busulfan	Mercaptopurine	A498	− 6.11
Azacitidine	Thiotepa	CAKI-1	− 16.33

## Results

NCI-ALMANAC contains 60 cell lines and 101 drugs. Although synonyms for drugs and cell lines exist in NCI-ALMANAC, in order to extract more abundant documents, we extracted synonyms for drugs and cell lines from PubChem and Cellosaurus, respectively. As a result, the number of words increased about 8 times, and a total of 13,962 words were extracted. We extracted 936,734 documents containing these words from PubMed and converted synonyms into their representative words. We extracted the features of drugs and cell lines based on the Word2vec algorithm using preprocessed documents. A total of 130,180 combinations of 60 cell lines and 68 drugs targeting at least one gene were used in NCI-ALMANAC [27]. Cell line features (expression, mutation, etc.) for the baseline were obtained from CellMinerCDB [28]. In addition, drug target and molecular features were obtained from DrugBank [29] using the RDKit package in Python. Various deep learning and machine learning algorithms were applied using the constructed drug and cell line features as training data.

**Fig. 2:**
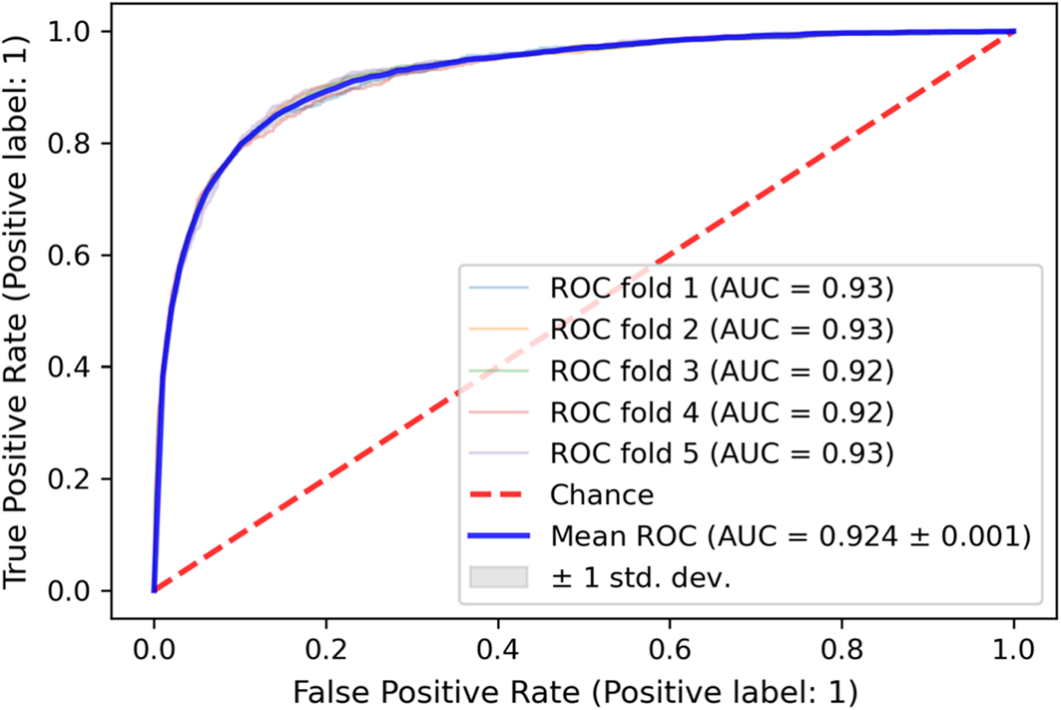
ROC curve of highest performing prediction model

Table 3 shows the performance model results according to the algorithm. We evaluated the prediction model performances using the unified framework by Kunjie et al. [27]. Their results were used as baseline, and the performance of the prediction model using our features was compared. The prediction model was evaluated five-times each with 5-fold cross validation. We evaluated the model performance using three indicators: area under the receiver operating characteristic curve (ROC-AUC), area under the precision–recall curve (AUPR) and F1 score. Since we repeated five times to evaluate the prediction model, we used the mean and standard deviation as comparative indicators. The prediction model performance constructed using only our features was higher than the majority of the baseline. Figure 2 shows the ROC curve of the FFNN-based prediction model, which is the best performance in ROC-AUC. Based on these results, we can infer that our feature extraction method was able to extract more drug and cell line information than the baseline method.

**Table 3:** Performance comparison of prediction models

Algorithms	FFNN		AE		XGB		ERT	LR
Ref.	[9]	[10]	[11]	[12]	[13]	[14]	[15]	[16]
(a) Performance comparison using ROC-AUC								
Baseline	0.912 ± 0.004	0.91 ± 0.005	0.914 ± 0.006	0.895 ± 0.007	0.885 ± 0.01	0.895 ± 0.005	0.843 ± 0.007	0.847 ± 0.006
Ours	**0.924** ± **0.001**	0.915 ± 0.006	0.923 ± 0.003	0.92 ± 0.004	0.889 ± 0.003	0.892 ± 0.004	0.881 ± 0.004	0.854 ± 0.007
(b) Performance comparison using AUPR								
Baseline	0.402 ± 0.017	0.417 ± 0.032	0.408 ± 0.025	0.339 ± 0.026	0.349 ± 0.016	0.381 ± 0.026	0.24 ± 0.015	0.192 ± 0.011
Ours	0.434 ± 0.014	0.427 ± 0.025	**0.438** ± **0.008**	0.424 ± 0.011	0.371 ± 0.013	0.381 ± 0.016	0.326 ± 0.012	0.196 ± 0.004
(c) Performance comparison using F1 score								
Baseline	0.359 ± 0.027	0.262 ± 0.045	0.325 ± 0.039	0.224 ± 0.05	0.227 ± 0.01	0.24 ± 0.019	0.272 ± 0.018	0.059 ± 0.01
Ours	0.392 ± 0.017	0.313 ± 0.052	0.296 ± 0.021	**0.428** ± **0.028**	0.271 ± 0.016	0.259 ± 0.02	0.263 ± 0.015	0.064 ± 0.013

## Conclusions

We introduced an approach to predicting the synergy of anti-cancer drug combination by using features extracted from documents. Our approach yielded higher prediction performance than the majority of published studies under the same conditions. Our approach makes two contributions to drug combination prediction.

As new drugs are discovered, the number of drug combinations increases dramatically. In practice, testing all drug combination cases proves difficult. Our approach can predict new anti-cancer drug combination synergy, reducing the time and effort invested. In other words, we expect that it would be a useful approach for researchers to predict new anti-drug combination synergy before in-vivo or in-vitro experiments.

Structuring the knowledge from newly published studies is time consuming because it is difficult to conduct without domain experts. Using unstructured data to extract features, our approach could be easily and immediately applied to new research. An additional benefit of our feature extraction method is that the extracted features can be utilized together with features from structure data.

Currently, the present model is exclusive to PubMed abstracts. We used PubMed because it is the largest repository of biomedical publication. In future works, the feature extraction method can be expanded to use more information by including full documents provided by PubMed Central, bioRxiv, and other databases. In the present model, we only extracted the drug and cell line features in NCI-ALMANAC. In future works, we will cover more anti-cancer drug combinations, including various large-scale public anti-cancer drug combination databases such as DrugComb [30] and DREAM [31]. Finally, our approach could also be used for other purposes. For example, by changing the input data, we can use it to predict the interaction between drugs and genes, and also the effects of drugs and diseases.

## Data Availability

The result is available at https://github.com/YongsunShim/anticancer-drug-combination-prediction-using-documents-based-features.git.

## Abbreviations

NCI: The National Cancer Institute
ALMANAC: A large matrix of anti-neoplastic agent combinations
FFNN: Feed-forward neural network
AE: Autoencoder
XGB: XGBoost
ERT: Extremely randomized trees
LR: Logistic regression
ROC-AUC: Area under the receiver operating characteristic curve
AUPR: Area under the Precision–Recall curve
DREAM: AstraZeneca-Sanger drug combination prediction DREAM challenge
